# Impact of Oral Lichen Planus on Quality of Life: A Cross-Sectional Study

**DOI:** 10.1055/s-0045-1811214

**Published:** 2025-08-26

**Authors:** Soulafa Almazrooa, Waleed Alamoudi, Sara Akeel, Sarah Ali, Nada Binmadi, Sana Alhamed, Ghada Mansour, Nada Alhindi, Doha Aljeddawi, Reem Bashammakh, Osama Felemban, Hani Mawardi

**Affiliations:** 1 Department of Oral Diagnostic Sciences, Faculty of Dentistry, King Abdulaziz University, Jeddah, Saudi Arabia; 2 Pediatric Dentistry Department, Ministry of Health, Madinah, Saudi Arabia; 3 Department of Orthodontics, Faculty of Dentistry, King Abdulaziz University, Jeddah, Saudi Arabia; 4 Department of Pediatric Dentistry, Faculty of Dentistry, King Abdulaziz University, Jeddah, Saudi Arabia

**Keywords:** oral lichen planus, quality of life, social change, psychometrics

## Abstract

**Objectives:**

Lichen planus is a chronic, mucocutaneous inflammatory disorder that often affects oral tissues. It can cause discomfort and pain in affected individuals, thereby affecting their daily lives and ability to function. This study aims to comprehensively evaluate the impact of oral lichen planus (OLP) on the quality of life (QoL) of a cohort of patients.

**Materials and methods:**

This was a questionnaire-based, cross-sectional study of patients with OLP attending the Oral Medicine Clinic, Faculty of Dentistry, King Abdulaziz University, Jeddah, Saudi Arabia. Demographic data and disease activity score were collected, and eligible subjects were invited to complete the Chronic Oral Mucosal Disease Questionnaire, Oral Health Impact Profile, and Social and Readjustment Rating Scale.

*Statistical analyses* were performed using the Statistical Package for the Social Sciences (IBM SPSS Statistics for Windows).

**Results:**

Thirty-eight participants completed the study, 27 of whom were females with an average age of 52.2 years. The average OLP severity score was 21.3 (range: 3–49). Prior to OLP, most of the participants experienced life events amounting to either moderate (44.7%) or high stress (28.9%). Overall, 74% of patients with OLP experienced discomfort during oral hygiene routines and OLP-limited oral hygiene practices in 58% of participants. In addition, 92% experienced variable degrees of discomfort with specific types of food (e.g., spicy food) and 70% of study subjects required medications to manage OLP, which helped 90% of patients. Moreover, 75 and 85% of the patients were emotionally and socially affected by OLP, respectively, experiencing stress and anxiety.

**Conclusion:**

Active and symptomatic OLP substantially impacts QoL, and stressful life events could trigger its development.

## Introduction

Lichen planus (LP) is a chronic, mucocutaneous inflammatory disorder that most commonly affects females aged between 40 and 50 years and often affects oral tissues (oral lichen planus [OLP]). 1 LP has a reported prevalence of 0.3 to 1.8% in Saudi Arabia and 0.5 to 2.6% globally. 1 2 3 4 5 Although pathologically benign, it causes a diverse set of symptoms of varying severity during its course of remission and exacerbation. 6 As such, LP can cause considerable discomfort and pain in affected individuals, thereby affecting their daily lives and ability to function. 7 Daily functions such as eating, drinking, and speaking may be problematic for patients with OLP. Its management includes, but is not limited to, topical and/or systemic immunosuppressive regimens including corticosteroids (CS), tacrolimus, and anti-inflammatory medications such as hydroxychloroquine. 6 However, a subset of patients may be unresponsive to these therapies or relapse despite treatment, challenging a favorable long-term prognosis.

Individuals with persistent or refractory symptomatic OLP can experience a decline in their quality of life (QoL), including disrupted eating, speaking, and oral hygiene practices. 7 These impairments may lead to chronic stress, fatigue, or depression. 7 Comprehensively understanding the impact of OLP on QoL requires a systematic evaluation of psychological well-being, ideally seamlessly integrated into holistic patient treatment planning and management. Various tools are available for assessing the QoL and psychological impact of chronic oral diseases. Among these, the Oral Health Impact Profile-14 (OHIP-14) and visual analog scale (VAS) have often been used, offering validated outcomes; however, these tend to be less sensitive for reporting minimal changes associated with OLP. 8 9 Recently, the Chronic Oral Mucosal Disease Questionnaire (COMDQ) was developed and assessed in diverse populations as a reliable and specific tool for psychometric testing in oral medicine, 9 and complementing nonspecific OHIP-14 data with tools like COMDQ can improve assessment. In addition, the Social and Readjustment Rating Scale (SRRS) is a useful tool for assessing stressful life events and is used extensively in the literature. 10 Combining the SRRS with COMDQ and OHIP-14 may provide an optimal method for evaluation.

Numerous studies have reported increased stress, anxiety, and other psychological disorders associated with OLP symptoms. 11 12 13 However, there is a lack of prospective studies in literature on the influence of OLP on daily routines and function in the Saudi population. We, therefore, evaluated the impact of OLP on patients' QoL in a tertiary medical center in Jeddah, Saudi Arabia, utilizing comprehensive, population-specific patient management to improve QoL outcomes.

## Materials and Methods

This was a questionnaire-based, descriptive cross-sectional study of an OLP patient cohort attending the Oral Medicine Clinic at King Abdulaziz University, Faculty of Dentistry (KAUFD), Jeddah, Saudi Arabia, between January 2018 and January 2024. Ethical approval was granted by the research ethics committee of KAUFD (#133-11-18). Written informed consent was obtained from all participants. The inclusion criteria were all adult patients aged ≥18 years with clinically diagnosed symptomatic OLP or those diagnosed clinically along with histopathological assessment following modified World Health Organization (WHO) criteria, excluding any other underlying medical conditions. 6 Exclusion criteria were patients with asymptomatic OLP, evidence of oral epithelial dysplasia, patients with both oral and skin LP, and those diagnosed with other mucosal conditions that could potentially induce chronic oral pain or psychological disorders such as burning mouth syndrome or persistent idiopathic facial pain. Patients on chronic pain management or antipsychotic/antidepressive medications were also excluded.

Demographic data on study participants were collected. For the purpose of this study, the oral disease severity score by Escudier et al was used to evaluate disease severity following a comprehensive oral examination. 14 This scoring system assessed 17 sites in the oral cavity for degree of involvement (0–2), severity (0–3), and disease activity (0–3) together with pain (0–10) over the last 2 weeks, providing a possible total score of 106.

Eligible subjects were asked to complete COMDQ, a psychometric questionnaire validated in English and Arabic and consisting of 26 items categorized into four domains: (1) pain and functional limitations; (2) medication and treatment; (3) social and emotional status; and (4) patient support. 9 The questionnaire was provided in both Arabic and English based on individual preferences. A score was derived for each respondent by aggregating replies based on the following scale: not at all = 0, slightly = 1, moderately = 2, considerably = 3, or extremely = 4, with scoring reversed for some items as per the questionnaire guidelines.

To assess QoL, participants were asked to complete the OHIP-14 questionnaire, which contains 14 items grouped into seven domains: (1) functional limitation, (2) physical pain, (3) psychological discomfort, (4) physical disability, (5) psychological disability, (6) social disability, and (7) handicap. 15 Response options were numerical on a Likert scale, ranging from never “0” to very often “4.” An OHIP-14 score was derived for each subject by summing the scores for each question.

The SRRS was used to assess the impact of life events on OLP ( Table 1 ). 10 This questionnaire consisted of 43 validated stressful life events experienced over the last 12 months, in which each item is given a score. For example, life events included positive events such as marriage, reconciliation with a spouse or partner, pregnancy, outstanding achievements, and going on holiday, while negative events included experiencing death in the family, marriage-related problems, personal injury, and health changes in a family member. Participants were asked to report the occurrence of these events before or after OLP onset. The total SRRS score for each subject was calculated, where a score of less than 150 indicated low stress and 30% risk of developing a stress-related illness; a score of 150 to 299 indicated moderate stress and a risk of 50% of developing a stress-related illness; and a score of 300 or more signified high stress and that the subject had an 80% chance of developing a stress-related illness. 10

**Table 1:** The Social and Readjustment Rating Scale (SRRS) to assess stressful life events

Death of spouse or partner
Divorce
Marital or partner separation
Time in prison
Death of a close family member
Personal injury or illness
Marriage
Fired at work
Got back together with partner or spouse
Retirement
Change in the health of a family member
Pregnancy
Sex difficulties
Gain of a new family member
Change to business
Change in financial state
Death of a close friend
Change to a different line of work
Change in the number of arguments with spouse or partner
Missed payments on the mortgage or loan
Change of responsibilities at work
Son or daughter leaving home
Trouble with in-laws
Outstanding personal achievement
Partner begins or stops work
Begin or end school
Change in living conditions
Revision of personal habits
Trouble with boss
Change in work hours or conditions
Change in residence
Change in child's school
Change in hobbies/pastimes
Change in church activities
Change in social activities
Change in sleeping habits
Change in number of family get-togethers
Change in eating habits
Holiday
Minor violations of the law

We hypothesized that OLP would have an impact on QoL and perceived stress and may be associated with life events. The primary outcome of this study was QoL in patients with OLP indicated by COMDQ and OHIP-14 scores.

### Statistical Analysis

Statistical analyses were performed using the Statistical Package for the Social Sciences (IBM SPSS Statistics for Windows, v20.0; IBM Statistics, Armonk, New York, United States). All collected responses were analyzed and presented as mean and standard deviation or frequencies and percentages.

## Results

Eighty OLP patients visited the Oral Medicine clinic at KAUFD, of whom 38 were eligible and were included in the study. Overall, there were 27 (71.1%) females, 11 (28.9%) males, with an average age of 52.2 years (range: 25–80). The average OLP severity score for study participants was 21.3 (range: 3–49).

### Quality of Life Assessed by COMDQ

Responses to COMDQ are shown in Table 2 . The mean COMDQ score was 58.4 ± 20.9. Regarding functional limitations, 74% of patients experienced discomfort during oral hygiene routines, and oral hygiene practices were limited in 58% of patients. Nearly all (92%) patients experienced variable degrees of discomfort with specific food types (e.g., spicy food). Consequently, over 80% limited the type and texture of food they regularly consumed, and over 30% were affected by changes in food temperature. Overall, 70% of study subjects required medications to manage OLP, which helped 90% of patients to varying degrees. Approximately 79% were concerned about the side effects of these medications, and 70% were frustrated that there was no specific cure for OLP.

**Table 2:** Participant responses to the Chronic Oral Mucosal Disease Questionnaire (COMDQ)

		Not at all a (%)	Slightly (%)	Moderately (%)	Considerably (%)	Extremely (%)	NA(%)
Pain and functional limitation	1. How much do certain types of food/drink cause you discomfort (spicy food, acidic food)?	7.9	7.9	23.7	28.9	31.6	0
	2. How much does lichen planus cause you to limit the types of food/drinks you consume?	18.4	23.7	21.1	18.4	18.4	0
	3. How much do certain food textures cause you discomfort (rough food, crusty food)?	18.4	7.9	18.4	28.9	23.7	2.6
	4. How much does lichen planus cause you to limit the textures of the food you consume?	18.4	13.2	28.9	18.4	18.4	2.6
	5. How much does the temperatures of certain foods/drinks cause you discomfort?	15.8	10.5	28.9	23.7	21.1	0
	6. How much does lichen planus cause you to limit the temperature of the foods/drinks you consume?	28.9	13.2	18.4	15.8	23.7	0
	7. How much does lichen planus lead to discomfort when carrying out your daily oral hygiene routine (brushing, flossing, mouthwash usage)?	26.3	18.4	18.4	18.4	18.4	0
	8. How much does lichen planus cause you to limit your daily oral hygiene routine (brushing, flossing, mouthwash usage)?	42.1	15.8	13.2	13.2	15.8	0
	9. How much does lichen planus lead to discomfort when wearing a denture (false teeth)?	26.3	2.6	5.3	2.6	63.2	0
Medication and treatment for lichen planus (including mouthwashes, gels, creams, ointments, injections, tablets, infusions)	10. How much do you feel you need medication to help you with activities of daily life (talking, eating, etc.)?	28.9	18.4	10.5	10.5	26.3	5.3
	11. How satisfied are you with the medication being used to treat lichen planus? a	10.5	15.8	23.7	10.5	7.9	31.6
	12. How concerned are you about the possible side effects of the medications used to treat lichen planus?	21.1	7.9	28.9	15.8	10.5	15.8
	13. How much does it frustrate you that there is no single standard medication to be used in lichen planus?	28.9	10.5	13.2	13.2	26.3	7.9
	14. How much does the use of the medication limit you in your everyday life (routine/the way you apply or take your medications)?	39.5	15.8	18.4	5.3	2.6	18.4
	15. How much does it bother you that there is no cure for lichen planus?	21.1	13.2	2.6	18.4	39.5	5.3
Social and emotional	16. How much does lichen planus get you down?	23.7	26.3	15.8	21.1	13.2	0
	17. How much does lichen planus cause you anxiety?	15.8	21.1	26.3	13.2	23.7	0
	18. How much does lichen planus cause you stress?	26.3	21.1	21.1	15.8	15.8	0
	19. How much does the unpredictability of lichen planus bother you?	13.2	15.8	18.4	36.8	15.8	0
	20. How much does lichen planus cause you to worry about the future (spread of the condition, possible cancer risk)?	10.5	15.8	18.4	28.9	26.3	0
	21. How much does lichen planus make you pessimistic about the future?	21.1	26.3	28.9	7.9	15.8	0
	22. How much does lichen planus disrupt social activities in your life (social gatherings, eating out, parties)?	52.6	13.2	18.4	13.2	2.6	0
Patient support	23. How satisfactory do you consider the information available to you regarding lichen planus? a	7.9	5.3	23.7	31.6	28.9	2.6
	24. How satisfied are you with the level of support and understanding shown to you by family regarding lichen planus? a	10.5	7.9	13.2	21.1	44.7	2.6
	25. How satisfied are you with the level of support and understanding shown to you by friends/work colleagues regarding lichen planus? a	13.2	10.5	10.5	23.7	36.8	5.3
	26. How isolated do you feel as a result of lichen planus? a	60.5	13.2	18.4	5.3	2.6	0

Abbreviation: NA, not applicable.

a Questions in which the response scale was reversed: not at all = 4; slightly = 3; moderately = 2; considerably = 1; extremely = 0.

Overall, 75 and 85% of patients were emotionally and socially affected to varying degrees by OLP, respectively, experiencing stress and anxiety. The majority (90%) of participants were troubled to some degree by the unpredictability of the disease and the risk of developing secondary oral cancer. Overall, OLP affected social activities and interactions with others in about half of patients, including social gatherings, attending parties, or eating out. Furthermore, 79% of subjects felt pessimistic about the future. Over 80% of subjects received support and understanding from family, friends, and work colleagues, which was helpful. On the other hand, 10% encountered inconsiderate individuals and 40% felt isolated to some degree ( Table 2 ).

### Quality of Life Assessed by OHIP-14

Detailed OHIP-14 responses are shown in Table 3 . The mean OHIP-14 score was 31.9 ± 6.7. In terms of stress experienced by symptomatic OLP patients, around 70% reported feeling nervous and stressed and upset by unexpected events. Of the study subjects, 20% could not effectively cope with changes in their lives, while 10% lacked the confidence to handle daily problems. Prior to developing OLP symptoms, 50% of subjects struggled to cope with all their responsibilities, and ∼70% of patients experienced anger over events beyond their control. Approximately 80% of patients were preoccupied with how to spend time and accomplish tasks before developing OLP symptoms, and >50% of patients felt overwhelmed by difficulties they could not overcome.

**Table 3:** Participant responses to the OHIP-14

Questions	Never (%)	Almost never (%)	Sometimes (%)	Fairly often (%)	Very often (%)
1. Prior to oral lichen planus symptoms, how often have you been upset because of something that happened unexpectedly?	18.4	13.2	26.3	28.9	13.2
2. Prior to oral lichen planus symptoms, how often have you felt that you were unable to control the important things in your life?	34.2	13.2	18.4	7.9	26.3
3. Prior to oral lichen planus symptoms, how often have you felt nervous and stressed?	13.2	18.4	26.3	21.1	21.1
4. Prior to oral lichen planus symptoms, how often have you dealt successfully with irritating life hassles?	2.6	5.3	34.2	23.7	34.2
5. Prior to oral lichen planus symptoms, how often have you felt that you were effectively coping with important changes that were occurring in your life?	5.3	13.2	23.7	34.2	23.7
6. Prior to oral lichen planus symptoms, how often have you felt confident about your ability to handle personal problems?	0	10.5	23.7	36.8	28.9
7. Prior to oral lichen planus symptoms, how often have you felt that things were going your way?	13.2	15.8	31.6	23.7	15.8
8. Prior to oral lichen planus symptoms, how often have you found that you could not cope with all the things you had to do?	21.1	28.9	34.2	2.6	13.2
9. Prior to oral lichen planus symptoms, how often have you been able to control irritations in your life?	15.8	18.4	18.4	21.1	26.3
10. Prior to oral lichen planus symptoms, how often have you felt that you were on top of things?	10.5	7.9	26.3	39.5	15.8
11. Prior to oral lichen planus symptoms, how often have you been angered because of things that happened that were out of your control?	15.8	15.8	23.7	18.4	26.3
12. Prior to oral lichen planus symptoms, how often have you found yourself thinking about things that you had to accomplish?	5.3	13.2	21.1	28.9	31.6
13. Prior to oral lichen planus symptoms, how often have you been able to control the way you spend your time?	5.3	10.5	15.8	44.7	23.7
14. Prior to oral lichen planus symptoms, how often have you felt difficulties were piling up so high that you could not overcome them?	31.6	10.5	34.2	7.9	15.8

### Associations between OLP and Stressful Life Events

The SRRS was used to collect data on stress-inducing life events both before and/or after the development of OLP ( Table 4 ). Prior to OLP, most of the participants experienced life events amounting to either moderate stress (44.7%) or high stress (28.9%). Following OLP, the majority of participants (73.7%) reported the occurrence of life events indicating low stress levels.

**Table 4:** Categorization of participants according to the Social Readjustment Rating Scale linked to OLP onset

*N* (%)	Before OLP	After OLP
Low stress (score < 150)	10 (26.3)	28 (73.7)
Moderate stress (scores 150–299)	17 (44.7)	8 (21.1)
High stress (scores 300 or more)	11 (28.9)	2 (5.3)

Abbreviation: OLP, oral lichen planus.

### Association between OLP Severity and QoL

Subjects were classified according to their severity ratings into low severity (lower 50%) and high severity (upper 50%). The mean COMDQ score in the low severity group (35.8 ± 23.2) was significantly lower than that in the high severity group (49.1 ± 16.7), with a statistically significant difference ( *p* = 0.049). Concerning QoL assessed by OHIP-14, no significant differences were seen between the two groups ( *p* = 0.459; Table 5 ).

**Table 5:** Effects of OLP severity on QoL

	Low OLP severity scores	High OLP severity scores	*p* -Value
COMDQ score	35.8 ± 23.2	49.1 ± 16.7	0.049
OHIP-14 score	31.3 ± 5.8	32.3 ± 5.9	0.459

Abbreviation: OLP, oral lichen planus.

Note: Mann–Whitney *U* -test.

## Discussion

OLP is a T cell-mediated condition that takes various clinical forms including reticular, erosive, ulcerative, and/or plaque-like changes. 1 Predominantly affecting women, OLP tends to induce pain, sensitivity, and textural changes in the oral tissues. 1 Persistent symptoms can fluctuate and variably impact eating, speaking, and the daily routine of affected individuals, potentially leading to anxiety, fatigue, and often fear of malignant transformation. 11 First-line treatment for OLP includes topical therapy with or without systemic CS therapy. 6 Upon achieving remission, other therapies such as tacrolimus, hydroxychloroquine, isotretinoin, and emerging treatments like etanercept, aloe vera, extracorporeal phototherapy, and mycophenolate mofetil can be considered, which aim to mitigate long-term CS-associated toxicities. 1 16 17 18 However, treatment side effects or a lack of response may exacerbate the psycho-emotional well-being of patients, highlighting the importance of assessing QoL as an integral component of overall OLP management.

The potential relationship between LP and psychiatric profiles is bidirectional. Anxiety, depression, or stress may influence the severity of LP symptoms, while longstanding symptomatic LP can also precipitate psycho-depressive disorders. 11 12 19 20 However, most of the available literature focuses on the QoL of LP patients in general and not specifically those with OLP. 21 22 The literature on LP and especially OLP is highly heterogeneous, with different populations and evaluation methods used, making direct comparisons with existing evidence challenging. Overall, 7 to 53% of LP patients are likely to experience moderate-to-severe depression and other psychological disorders. 20 These figures might differ in the OLP population, considering the impact of affected sites. A study done on 100 patients with LP established that LP affected QoL in 78% of cases. Of these cases, 42% had oral manifestations. 23 These patients were evaluated with different indices: Dermatology Life Quality Index (DLQI) and the EuroQol five-dimensional three-level score. Additionally, the depression symptoms were evaluated using Beck Depression Inventory II. Twenty-nine percent of patients had mild-to-moderate symptoms of depression, and 6% had severe symptoms of depression. Patients with genital LP had the highest effect on their QoL. 23

Depressed mood, low self-control, anxiety, and low QoL were shown to have a high association with OLP. 24 25 26 Stress, anxiety, and depression were evaluated in OLP patients and compared to negative and positive controls using the General Health Questionnaire-version 28 (GHQ-28) and the Hospital Anxiety and Depression Scale (HADS), which showed a significant difference when compared to the negative control and no significant difference to positive control group. This indicated a strong association between OLP and stress, anxiety, and depression. 27 A study comparing 80 OLP patients to a control group in 2003 reported high cortisol levels in the saliva of OLP patients as well as high anxiety using Spielberger's State-Trait Anxiety Inventory. 28 A study by Wiriyakijja et al recruited 300 OLP subjects and assessed their QoL. 29 They concluded that OLP-associated pain, anxiety, and stress were significantly associated with QoL. A recent systematic review of 17 studies assessed QoL in OLP patients measured by the OHIP-14, and determined that OLP had a moderate impact on QoL. 30 Nevertheless, disease control improved QoL over the longer term. As an important aspect of QoL, Hampf et al reviewed the mental health of patients with OLP and reported that 21.4% had minor mental illness, 5.4% had moderate mental illness, and 25% had severe mental illness. 31 Consequently, mental stress exacerbated OLP symptoms. Therefore, psychological/psychiatric support should be considered as part of the overall management plan of OLP.

The management of OLP is typically tailored to each patient based on their symptoms, if present. In general, asymptomatic and keratotic OLP require patient education and reassurance alone. Patients with oral symptoms may receive topical and/or systemic treatment to provide comfort and improve their QoL. In this study, 70% of patients needed medications to manage their OLP symptoms and to help with daily life activities, with more than 90% response rate. Even with concerns about medication-induced toxicities, patients continued to use these agents based on needs and disease symptoms. This observation is consistent with that of Radwan-Oczko et al, who reported poorer QoL in patients with a long duration of symptoms, leading to higher stress levels. 32 Kengtong in 2023 demonstrated improvement in the QoL in 72 Thai patients after 1-month treatment with topical steroids using the Oral Impact on Daily Performances index (OIDP), which assessed eating, sleeping, speaking, smiling, and carrying out major work through a questionnaire, and Patient Global Impression of Change (PGIC). 33 Approximately 54% of the patients greatly improved and 39% moderately improved. 33 Moreover, Mahon-Smith et al performed a qualitative analysis of patients with OLP and its effect on daily activities such as oral hygiene, physical function (chewing, swallowing, and mouth movements), and emotional well-being (frustration, embarrassment, sadness, worry, and being unable to perform social activities). 34 In the current study, 90% were affected by the unpredictability of the disease and the risk of developing cancer. Furthermore, OLP affected social activities and interactions with others, such as at social gatherings, attending parties, or eating, in 50% of participants, and 40% felt isolated and pessimistic about the future. Controlling OLP symptoms and pain was helpful for alleviating anxiety and discomfort, potentially alleviating stressors, reducing the risk of depressive disorders, and enhancing overall QoL.

Life events that cause stress, anxiety, and depression can trigger OLP symptoms. 35 Psychosocial stresses are also known to induce autoimmune and inflammatory conditions, leading to psychosomatization. 36 These events are typically assessed via validated questionnaires to attain the correct diagnosis, staging, and management plan. 37 In the current study, we reported that 75 and 85% of participants suffered from stress and anxiety, respectively. Prior to OLP, most of the participants experienced life events amounting to either moderate stress (44.7%) or high stress (28.9%), which could have triggered OLP. A recent study by Alnazly et al described a significant association between OLP symptoms and stress, anxiety, and depression. 11 Recently, salivary biomarkers have been proposed as a potential diagnostic tool for the diagnosis of various psychological events. 37 For instance, a recent study by Simoura et al reported a relationship between stress in OLP patients and a decrease in salivary alpha-amylase levels. 35 37 38

We characterized the association between OLP symptoms/severity and QoL, including the psychological profiles of affected individuals. No other study has assessed the impact of OLP on QoL in Saudi Arabia. Nevertheless, this study has some limitations. Even though the sample size was collected over a long period of time, it was relatively small due to the stringent criteria, length of questionnaires, and sensitive nature of the topic. While the study recruited participants from a regional referral center, it is still a single-site design, which may limit the generalizability of the results. In addition, there was no control group for intergroup comparison, which was hard to recruit due to the length of the assessment and the sensitivity of the topics discussed in the assessment. Furthermore, all the assessments relied solely on subjective questionnaires. It was not reinformed by clinical examination and OLP scoring after treatment. Nevertheless, the study validates previous findings and emphasizes the significance of psychological evaluation and intervention in the overall management plan for patients with OLP.

## Conclusion

Active and symptomatic OLP may substantially impact the QoL of affected individuals. In addition, stressful life events, whether pleasant or not, could trigger the development of OLP. Dental practitioners must be aware of the psycho-pathological effects of OLP to formulate an optimal management plan. Further longitudinal studies are imperative to validate these findings and provide a more comprehensive understanding of the relationship between OLP and its psychological consequences.
